# “The Rest of my Childhood was Lost”: Canadian Children and Adolescents’ Experiences Navigating Inflammatory Bowel Disease

**DOI:** 10.1177/10497323211046577

**Published:** 2021-11-24

**Authors:** Claudia Barned, Alexis Fabricius, Alain Stintzi, David R Mack, Kieran C O’Doherty

**Affiliations:** 1 UHN Bioethics Program, 7989 University Health Network, Toronto, Canada; 2 Pragmatic Health Ethics Research Unit, 5598 Institut de recherches Cliniques de Montreal, Montreal, Canada; 3 Department of Psychology, 3653 University of Guelph, Guelph, Canada; 4 Ottawa Institute of Systems Biology and Department of Biochemistry, Microbiology and Immunology, Faculty of Medicine, 6363 University of Ottawa, Ottawa, Canada; 5 Children’s Hospital of Eastern Ontario (CHEO) IBD Centre and Department of Pediatrics, 27338 University of Ottawa, Ottawa, Canada

**Keywords:** chronic illness management, pediatric illness experiences, qualitative health research, inflammatory bowel disease, patient education resources

## Abstract

Children and adolescents with Inflammatory Bowel Disease (IBD) face significant and unique challenges related to their condition. The aim of this study was to better understand some of these challenges, and to explore how Canadian youth respond to them. We interviewed 25 pediatric patients with IBD, ranging in age from 10–17, to find out about their illness experiences. Using a thematic analysis, we discerned three themes: *challenges related to diagnosis*, *making sense of change*, and *navigating sociability*. Taken together, they paint a picture of young people facing great uncertainty prior to diagnosis, pronounced changes to selfhood as they make lifestyle adjustments, and facing difficulties with the implications of reduced sociability because of their disease. We conclude by providing recommendations for the development of resources aimed at helping newly diagnosed pediatric patients navigate these issues.

## Background

Inflammatory Bowel Disease (IBD) is an incurable chronic gastrointestinal (GI) disorder that results in recurrent acute and chronic inflammation of the GI tract. The two major subtypes of IBD include Crohn’s disease (CD) and ulcerative colitis (UC), both of which may cause extreme fatigue, recurring (often bloodied) diarrhea, rectal bleeding, abdominal cramps, stunted growth, appetite loss, and weight loss (Micallef–Konewko, 2013; Nicholas et al., 2007, 2008). Given the stigmatized nature of these symptoms, IBD is associated with a host of psychosocial issues, including anxiety, stress, loneliness, and depression (Jelenova et al., 2016; Loftus et al., 2011). Individuals with IBD face a condition marked by unpredictability, including intermittent “flares” when symptoms become active. In some cases, inflammatory responses can be addressed with diet changes and/or medication, though the latter can be accompanied by unpleasant side effects (Massironi et al., 2013). For those who are unresponsive to medical therapies, surgical interventions remain a treatment option.

Up to a quarter of IBD cases begin during juvenescence (Benchimol et al., 2011); of the approximately 233,000 Canadians living with IBD, an estimated 5900 are youths (Crohn’s and Colitis Canada, 2018). Given Canada’s high rates of pediatric IBD (Benchimol et al., 2011), that children and adolescents’ experiences of this condition are known to diverge from adults’ in unique ways (Brydolf & Segesten, 1996; Karwowski et al., 2009; Kluthe et al., 2018; Mamula et al., 2003), and that there is a comparative dearth of studies that consider their lived experience (Nicholas et al., 2007; Saunders, 2014), it is vital that we understand the nuances of children’s experiences, as well as how they navigate the challenges associated with this disease, from diagnosis to living with its chronicity.

## Pediatric IBD

Childhood and adolescence are known to be difficult developmental phases; with the added burden of a chronic illness like IBD, these periods can become marked by exaggerated or additional issues (Nicholas et al., 2007; Saunders, 2014).

### Physical Changes and Body Image

IBD is known to alter children’s appearance. As many as 65–85% of children with CD face growth failure and delayed puberty (Gasparetto & Guariso, 2014); in some cases, children can be up to two standard deviations below the norm in height (Vasseur et al., 2010). In Brydolf and Segesten’s (1996) qualitative study, participants described feeling embarrassed about both their symptoms and the physical changes associated with their medication; indeed, acne, loss of energy, weight loss, or rapid weight gain caused them to feel like they were “a stranger to oneself and different from others” (p. 45). Because youth place a high level of importance on their bodily appearance and physical attractiveness, these bodily changes can be difficult to process. Unsurprisingly, body image concerns have been raised in studies focusing on pediatric IBD experiences in varying countries and contexts (see Brydolf & Segesten, 1996; Daniel, 2002; Nicholas et al., 2007; 2008; Karwowski et al., 2009; Ruan et al., 2020). In some cases of chronic illness, such feelings can motivate extensive body projects to achieve a “normal” appearance (Balfe, 2009).

Youths who have had ostomy surgery face additional challenges, especially with respect to body image (Nicholas et al., 2008). A qualitative study by Nicholas et al., (2008) illustrated that some participants were “grossed out” by their post-operative body, though body image was further impacted by frequent incursions into the participants’ bodily space from frequent medical examinations and procedures. The presence of stoma bags has been shown to exacerbate feelings of difference and to generate (at least temporarily) resentment toward the body, which children and adolescents perceive as being “damaged” or “flawed” (Fourie et al., 2018; Nicholas et al., 2008; Savard & Woodgate, 2009).

### Psychosocial Issues

Unsurprisingly, these bodily changes and other challenges related to IBD tend to result in myriad psychosocial issues that are often underappreciated in clinical settings (Nicholas et al., 2007). For those who must undergo stoma surgery, having to live with a device attached to the skin into which feces are excreted can be distressing and exigent, both physically and emotionally (Jayarajah et al., 2016; Capilla–Diaz et al., 2019). This seems to be particularly true for youth who receive little post-operative psychological support (Polidano et al., 2021). Polidano and colleagues’ (2021) study noted that young IBD patients typically do not recognize their candidacy for such services, thus highlighting the need for physicians and health care workers to assign greater priority to identifying psychological supports for patients adjusting to their new life circumstances.

More broadly, however, experiences of pediatric IBD tend to produce feelings of embarrassment, loss of control, and stress with respect to enduring hospitalization, pain, extended diagnostic studies, dealing with medications, needing to stay near a bathroom, school absences, activity limitations, and isolation from others (Brydolf & Segesten, 1996; Daniel, 2002; Hall et al., 2005; Kleinman et al., 2004; Lindred et al., 2008; Micallef–Konewko, 2013; Nicholas et al., 2007; Savard & Woodgate, 2009). Though these issues can have adverse effects on self-esteem and socialization (Lindred et al., 2008), flare-related school absences are thought to be especially distressing (Moody et al., 1999). Not only are children concerned about lost time, underachievement, and their inability to partake in recreational activities at school, they may also sometimes experience additional stressors from unsympathetic teachers and peers (Moody et al., 1999). As Micallef–Konewko (2013) notes, “peers and teachers may find it difficult to comprehend that having IBD may prevent young people [from] doing things that others may do, for instance feeling too tired to stay up late and have fun, or having difficulty concentrating in class” (p.5).

Psychosocial issues can also emerge in response to the unpredictable nature of the disease, which can lead to embarrassing incidents of fecal incontinence at school or in public (Mamula et al. 2003). Saunders’ (2014) discourse analysis demonstrated that youths tend to highlight the stigmatizing nature of IBD, given the taboo quality of symptoms and the fact that aspects of the illness are certainly perceptible. Children often fear being singled out for needing to make frequent trips to the bathroom, for having to take medications or follow special diets while in school, for changes in their appearance and/or for having reduced capacity to participate in extracurricular or social activities (Alexakis et al., 2015; Mackner & Crandall, 2005; Nicholas et al., 2007, 2008). These anxieties can make it difficult for children and adolescents to talk about their illness, which can further deny them the opportunity to make sense of what is happening to their bodies or to receive support (Barned et al., 2016). Disclosure decisions are perceived as being fraught with risk, with youths often fearing and anticipating negative reactions (Carter et al., 2020; Daniel, 2002). Accordingly, secrecy is a common facet of pediatric IBD, as one way to control stigma is by electing to keep one’s health status clandestine (Hommel, 2013). This is especially true with respect to the presence of an ostomy, which can make disclosure decisions even more complex (Brydolf & Segesten, 1996; Daniel, 2002; Nicholas et al., 2007, 2008); in fact, the participants in one phenomenological study described feeling as though they were forced to hide their “true” self from others (Savard & Woodgate, 2009). This perceived need for secrecy can further engender feelings of alienation, isolation, and exclusion (Barned et al., 2016; Micallef–Konewko, 2013).

### Diet

Diet and mealtimes are important aspects of living with IBD. The effects of food on IBD symptoms and management, as well as its role in IBD-related mucosal inflammation, have been gaining scholarly interest. The participants in Chuong et al., (2019) qualitative study described having to implement numerous dietary restrictions for managing flares (e.g., avoiding or moderating certain foods (e.g., processed foods, following special diets)). For example, some children followed the specific carbohydrate diet (SCD) that removes grains, added sugar and milk products. While demonstrating success in reducing symptoms among pediatric patients, the SCD has also been associated with weight loss and isolation from peers due to the rigor required during mealtimes and stress in the parent–child relationship (Obih et al., 2016). Others rely exclusively on enteral nutrition, where special liquid formulas are ingested either by drinking or sometimes by means of nasogastric tube feeding. Both restrictive diets and enteral nutrition have been described as embarrassing for the fact that they make children and adolescents feel different from their peers (Kleinman et al., 2004).

### Family

Challenges extend to the family, as well. Children and adolescents report increased levels of personal attention, concern, and parental involvement, particularly when experiencing symptoms; while some children appreciate the attention, others resent the heightened scrutiny and describe feeling suffocated (Alexakis et al., 2015; Brydolf & Segesten, 1996; Nicholas et al., 2008). The participants in Nicholas et al., (2008) study recounted their parents being overly protective and consumed with worry. In some cases, however, family experiences can be quite different. Alexakis et al., (2015) UK-based qualitative study with Black and ethnic minority youth illustrated that families lacking culturally competent information or health services can unintentionally aggravate children’s experiences of IBD in key ways. For example, the authors found that some hide aspects of their illness to avoid upsetting their parents. In other cases, parents lacked an understanding of what IBD was or how to treat it, assuming that the condition occurred due to dietary choices; accordingly, participants described their parents purchasing food that, while nutritious, did not support symptom management and exacerbated difficulties with eating and managing symptoms.

These studies demonstrate how varied and complex pediatric IBD can be. For recently diagnosed youth, navigating IBD without informational supports adds to this complexity. Some foundations have begun developing patient education resources (see Crohn’s & Colitis UK, 2014, 2016; Crohn’s & Colitis Canada, 2017, 2018; Crohn’s & Colitis Foundation, 2021) to alleviate this burden; however, culturally nuanced materials are rare, as are public facing resources that focus specifically on the psychosocial aspects associated with navigating pediatric IBD in Canada. Canadian children and adolescents, particularly those from diverse cultural backgrounds are thus left wanting, as the current supports are primarily written for adults/parents/teachers and from a Western/Eurocentric lens.

We now turn to our own study, in which we explore the illness experiences of Canadian children and adolescents with UC and CD to discern not only what issues they face, but what lifestyle adjustments and strategies they devise in response to their circumstances. Lyons and Chamberlain (2005) remind us that the illness experience “always occurs in context” and that “ways of making sense of illness experience are a function of the social location of that person” (p. 285). As such, we recognize that patients’ experiences of an illness are situated, rather than universal; for example, experiences are affected by cultural context, socioeconomic status, geographical location, available treatments, the health care system being accessed, and current societal representations and stigma associated with the illness. Given that research on the lived experience of pediatric IBD is quite limited, it is important not to assume that existing studies represent the experiences of all individuals with IBD across these factors. Thus, the first objective of our study is to add to the small but growing number of studies exploring the lived experiences of youths with IBD, and to do so by considering qualitative nuances of their experiences located in particular social contexts. Our second objective is to provide suggestions for the development of resources aimed at newly diagnosed Canadian youths to help them navigate this disconcerting and stressful condition.

## Method

### Participants and Recruitment

During the months of February–May 2014, Barned conducted semi-structured interviews with 25 pediatric patients with CD (*n* = 17) and UC (*n* = 8), attending an IBD Center at a major children’s hospital in Ontario, Canada. The IBD Center is situated in the National Capitol region, and therefore serves patients hailing from eastern Ontario and western Quebec. This region has one of the highest median incomes and high levels of education in comparison to other regions in the country. In addition, the healthcare system ensures there are no costs associated with physician visits, hospital visits, tests, or procedures; prescription drug costs are commonly covered through private employer insurance or through a government-based program.

The clinical research nurse at the IBD Centre recruited participants prior to their scheduled appointment at the clinic, or at a time of the parents’ choosing that was separate from their appointment. In total, 64 participants were approached; two declined to participate, citing lack of interest and being in a rush. Other participants who expressed interest but ended up not participating offered reasons including tiredness, feeling ill, being unable to stay, lack of time, or no upcoming appointment. Participants included 13 boys and 12 girls, ranging in age from 10 to 17 years old (M = 13.8). From the interviews, it was evident that the participants’ differed in terms of the types of medication they were on, the types of symptoms that affected them, and even the age at which they were diagnosed, offering a range of experiences and perspectives for analysis.

### Procedure and Materials

The clinical research nurse introduced the participants to Barned on the day of their interview. Barned described the purpose of the study, explaining that participants were to be interviewed about their experiences living with IBD as part of a larger study exploring youth’s involvement in biomedical research (see Barned et al., 2016, 2018). Consent/assent forms were read aloud and signed. A semi-structured interview guide was developed to structure conversations; however, new topics and questions were explored as they emerged organically in interviews. Sample questions included, “What is life like with IBD?” “Can you tell me about your experiences before diagnosis and while seeking treatment?”

This study was conceived of as being about the lived experiences of children; however, some participants asked for their parent(s) or caregiver(s) to accompany them. In four of the 25 interviews, one or both of the participant’s parents were also present; in these cases, parents contributed to the interview by clarifying the researcher’s questions to the participant or by supplementing the child’s response with additional detail (e.g., reminding the child of particular events, symptoms, and names of medication). In rare cases, parents contributed more substantively than we had anticipated by providing information about the nature of the participant’s illness, and insight on how it affected their family. Parents were not formally enrolled as research participants in our study, and we recognize the ethical problems with quoting them in the analysis; however, our view is that excluding the contextual information they provided would jeopardize the quality of the work since it augments the participants’ statements in important ways. Moreover, the participants and their parents are in trusting relationships with each other, and their contributions demonstrated this mutual support toward each other. Therefore, we supplement the analysis in one section with information offered by both parents, but we do so without direct quotes; instead, we paraphrased their comments that directly pertain to the experiences of the child who had difficulty articulating these points and thus encouraged input from her parents.

All interviews were conducted in English, except for one that was partly conducted in French, with the participant’s mother helping with translation. Participants and their parents were assured that any information provided would be kept confidential. Interviews ranged in length from 25 min to 2 h, with most of the longer interviews being with adolescent participants, and the shorter interviews being with younger children. Participants/their parents received compensation in the form of parking passes valued at $13 CDN or a bookstore gift card when a parking pass was not appropriate. Interviews were audio-recorded and transcribed verbatim, with Barned listed as I for Interviewer in the transcripts and pseudonyms assigned. Ethics approval for this study was obtained from the research ethics boards of Children’s Hospital of Eastern Ontario (CHEO) and the University of Guelph.

### Data Analysis

We used a thematic analysis (Braun & Clarke, 2013) to identify patterns in participants’ talk. This method aims “to capture participants’ experiences and perspectives and grounds research in participants’ accounts, rather than researcher’s categories” (Clarke & Braun, 2014, p. 1947). Because this is a “bottom-up” approach to analysis, it is well suited for exploring a range of research questions, including those related to elucidating experiences of a phenomenon like illness. We followed Braun and Clarke’s (2013) process: (1) familiarization with the data by repeated readings of the transcripts; (2) generating initial codes that captured the participants’ challenges and adaptations; (3) collating similar codes into potential themes using a Word document, ensuring that we had extracts from all 25 participants; (4) reviewing potential themes; (5) we eventually chose, defined, and named our themes, while refining the analysis, and (6) writing up the study. During the analytical process, the transcripts and audio-recordings were checked frequently to verify the interpretations of participants’ statements. Minor changes were made to the content of the extracts only in instances where information could be used to identify participants. Across all stages of the analysis, we were guided by Braun and Clarke’s (2013) 15 criteria for ensuring quality in qualitative research.

## Results

We discerned three themes related to Canadian children’s challenges and consequent adaptations related to IBD: *challenges related to diagnosis, managing identity and making sense of change,* and *navigating sociability.* Taken together, these themes demonstrate the unique difficulties of living with an invisible, chronic disease at such a young age.

### Challenges Related to Diagnosis

Issues related to obtaining, understanding, and accepting an IBD diagnosis were raised by many participants. For some, the challenge was being in a prolonged state of unknowing, both with respect to lacking an understanding of what was happening to their bodies, as well as having to face medical professionals’ uncertainty while they tried to determine what the participants were dealing with. For others, diagnostic challenges had to do with not knowing what IBD was, as well as being unsure of what to expect. Accordingly, several participants reported feeling confused and uncertain around the time of their diagnosis. In the extract below, a female participant describes her first experience with Crohn’s disease and the time leading up to her diagnosis.

I got it when I was in grade 6. It was not a fun year, sometimes I considered it as kind of the rest of my childhood was lost because I was battling Crohn’s. My parents decided to put me in for a whole bunch of tests with my regular doctor and back then Crohn’s wasn’t as popular as it was now, so you didn’t really know how to diagnose it. So, we went into a lot of testing and then came in contact with Dr. X, and one look right away, he knew it was Crohn’s.

As evidenced by the participant’s description above, obtaining a diagnosis of pediatric IBD can be difficult; however, those difficulties are multi-faceted. First, the *time* taken to receive a formal diagnosis is notable, as it took a year. Second, the *nature* of this interval is important, as it was marked by confusion, hospital trips, numerous procedures, and encounters with professionals with varying levels of knowledge about Crohn’s disease. Third, the *meaning* the participant attaches to this phase is consequential, as she describes the pre-diagnosis period as a turning point in her own development. This period that was long, punctuated with uncertainties and characterized by the presence of a looming specter, represented such a pronounced disruption in her childhood that she declares her childhood “lost”; instead, she is forced to undergo early maturation as she battles against her disease.

In the case below, another female participant describes the challenges she faced at school in the period prior to her diagnosis, especially with respect to school officials. In this section of the interview, the participant’s parents contributed by providing important details about her experience with school officials, though we have paraphrased their speech below, as they did not formally participate.

The participant’s parents describe her as being barred from making multiple trips to the bathroom because she lacked a formal diagnosis; consequently, the participant would have accidents at school, leading to her being bullied by her peers. Because she used the bathroom 20-30 times per day, school officials said that the participant’s trips were inappropriate and disruptive to the class, as they assumed that she was acting out to miss school. Despite her parents’ requests for special accommodations, the school refused to allow the participant to use the bathroom frequently because they did not believe the participant or her parents’ claims about her condition. Consequently, the participant did not attend school for nearly two years. Though she tried to return for short stints, these efforts were often derailed by her exhaustion, or by her need to go home to use the bathroom. School officials eventually threatened to have the participant removed from her parents’ custody, as they believed she was faking her condition and having troubles at home.

P: That’s why I like being home because I get homeschooled then the bathroom’s right there.

Only after receiving a diagnosis did the school officials come to realize that the participant’s condition was genuine.

Here, issues stemming from a lack of diagnosis are quite different. Because pediatric IBD is relatively unknown among the lay population—including symptomology and types of accommodations required—the school officials were unwilling to oblige the participant’s requests without sufficient justification. In this case, the time it takes to secure a diagnosis is not just confusing, but also painful and traumatizing, ultimately forcing the participant to leave her school so that she can attend to her symptoms. Indeed, it is unsurprising that this participant expresses a preference for homeschooling in the absence of a diagnosis so that she does not have to account for her illness-related behaviors, nor does she have to address issues of trust from suspicious school officials or bullying from peers.

Challenges did not end once participants were diagnosed. On the contrary, new issues emerged, including the need to grapple with what it meant to be diagnosed with IBD as a young person. Another female participant describes her own struggles:

P: And then knowing, it was like a huge reality check that like there is a disea – it scared me more because it was like Crohn’s “disease” because most kids don’t expect to have a disease, you know.

I: So is it, the issue [was] around the word “disease”?

P: Right, like when you think *disease*, you think like a sick person in a hospital bed struggling for their life kind of thing… So to me, when they said, “Oh you have Crohn’s disease”, I’m like, “*Disease*?! What?”

P: But now that I have a disease, it’s like I’ve seen more the other side that you don’t have to be some sick person. Obviously you’ll go through stuff but like, when people look at me, they don’t say, “Oh she has Crohn’s disease” or “She has some disease”; they just see me as a normal person. But back then it was like I felt really different.

This extract highlights the dynamic nature of the participant’s meaning making as she endeavors to understand what it means to have IBD. When first diagnosed, the participant is forced to confront her own construction of the idea of *disease*, which she connects with impending death; naturally, she is distressed and confused when she is informed of her own diagnosis. As her experience with her condition progresses, her understanding of what it means to have a disease also changes; once realizing that her outward appearance or behaviors do not change much with IBD, nor is she afflicted by symptoms all the time, having a disease and living with it loses some of its salience. This suggests that at least part of this participant’s initial distress at diagnosis was caused by struggling to understand what it means to both have a disease, and to be known as someone with a disease.

### Managing Identity and Making Sense of Change

In this section, we explore the types of adaptations that the participants made in light of their diagnosis, and how they responded to those adaptations. Most participants described making numerous lifestyle adjustments once they started experiencing symptoms, and even more upon diagnosis and when beginning treatment. In general, adjustments mostly centered around modifications in diet and activities; however, depending on the nature and severity of the participant’s symptoms, unwelcomed changes in behavior and goals also occurred. In the extract below, a female participant describes the former.

P: Yes, it had quite an effect on me…like Prednisone, I really hate because it makes me have this stomach, I say it’s not my stomach because the medication did it to me, it makes me eat, eat, eat and eat and I’m always hungry… So, like I will have a big meal and then an hour later I will be starving again and looking for something else to eat because I’d be so hungry and the Prednisone just makes you eat, eat, eat, eat, nonstop. It makes me have these cheeks, too. And I’m really hoping they are gone before the end of the year because of my graduation pictures from elementary school.

This participant’s most concerning lifestyle change is her Prednisone-induced eating, which causes her to gain weight. Overeating is a behavior that is laden with implications; often, it is portrayed as being a moral issue that is within the individual’s control, such that weight gain is seen to be caused simply by a lack of concern for one’s health, irresponsibility or poor will power. However, overeating has gendered implications, too. Maintaining/attaining a thin body size has been constructed as a key component of Western health and beauty norms; one’s feminine identity is called into question when there are notable deviations. Gaining weight moves an individual further away from this ideal, with consequences not only for her attractiveness, but also for her femininity, and perceived health. The participant emphasizes the impact of the medication on her behavior (“it makes you eat eat”); most notably, she discursively separates herself from the actions motivated by the Prednisone by putting distance between herself and the moral implications of her behavior. She even goes as far as to disown the parts of her body that are most affected, and frames the medication (not herself) as being responsible. Responding to changes in her appetite and her body by explaining that “it’s not my stomach” is a way to manage her identity and to make sense of her bodily changes.

Participants also spoke of the sacrifices they were forced to make because of their condition. One female participant explains that IBD forced her to give up meaningful activities.

P: I go to X high school, and I went there for X program… that required a whole lot of my effort and time. So, I was always at practice and I would be at school really early in the morning and stay late for practice after school, so it was a really stressful program. Then I got diagnosed a week before grade twelve (so this year), and I struggled with that because the workload was too much, so I had to drop out of the program… that’s what I love and I just had to drop it and now I’m just kind of like progressively trying to get better and just pass high school. … Having to decide to drop out of the X program was probably one of the hardest things I’ve ever done, but also at that time, I was on Prednisone so I can’t really say how much of it was me and how much of it was the drug but it really, really upset me. I felt really lost because I’ve been doing [name of activity] since I was four years old. And it’s what I’ve always known…I kinda felt like everything I knew was kind of taken out from underneath my feet.

The participant establishes herself as an ambitious young woman in a demanding school program, who has a long history of being involved in sports. When her symptoms necessitate major lifestyle changes, such that she can no longer play her sport or even finish her school program, she is forced to adjust by stopping her training and downgrading her scholastic goal to simply passing high school. As someone who initially framed herself as being intellectually ambitious and athletic, these lifestyle changes mean that she can no longer think of herself as being sporty and excelling at school, which has major implications for her identity. Like the previous participant, who distanced herself from undesirable behaviors, this participant also separates herself from the actions that conflict with her perception of herself by pointing to the role that her medication may have played in this change. This response suggests that the participants are attempting to manage their identity as they struggle to understand who they are and what they can do once diagnosed with IBD.

### Navigating Sociability

Being social with IBD is difficult, and it can be even more challenging as a youth. Many participants talked about trying to navigate sociability, with varying degrees of success; some elected to engage in isolation, while others attempted to socialize in a restricted manner. In this extract, a male participant explains how his condition shaped his social life:

P: Colitis affects my liver. I can’t drink alcohol so that presents, well, it’s not really a problem, but I can’t do the stuff that some of my friends are doing. I can’t, I won’t ever be able to drink alcohol.

I: What were your thoughts when you heard that?

P: It made me kind of sad and annoyed, but I’ve kind of gotten over it. I realized you don’t really need it, and I probably will save a lot of money.

I: Of course. So what did your friends say when you told them?

P: Some people brush it off, like it’s not a big deal. They go like, “Oh, don’t worry; you don’t need alcohol to have fun.” And then some people are like, “Oh man, that sucks.” Because, I guess I can’t do what they are doing, sometimes; but, I can still go to parties, but I am not [experiencing the party on] the same level [they are].

The participant’s ulcerative colitis-associated condition prevents him from drinking alcohol, denying him the opportunity to partake in a shared activity that is often positioned by teenagers as being key to socialization and acceptance (see Coleman & Cater, 2005). Accordingly, his condition has implications for his sociability. While he tries to rationalize the benefits of his situation and claims to have “kind of gotten over it,” he explains that he continues to struggle with being unable to partake in the same kinds of activities as his friends, noting that even if he is present at the same party, he will be unable to experience it on “the same level.” Thus, the participant’s concern is not so much about not being able to drink alcohol, as it is about what not drinking alcohol *means* for him in a social setting, especially in relation to his friends. By highlighting that his restrictions preclude him from *ever* drinking alcohol, he resigns himself to a lifetime of socializing differently than his peers.

The way in which participants navigated such scenarios differed. For example, unlike the previous participant, another participant described excluding herself when faced with restrictions; in fact, she asked to leave school rather than be singled out for her enteral nutrition-based diet:

P: Well at first it affected a lot, because the treatment I had to do was Modulen^™^ treatment [a nutrition product] - a shake, kind of like ‘Ensure^™^’ except I had to drink 6 shakes a day and I couldn’t drink anything that wasn’t clear fluid for 2 months. So, I literally did not eat any food for 2 months but I was gaining a lot of weight so that changed a lot. I convinced them to take me out of school because I did not want to eat lunch with people just drinking a shake and having to drink shakes in class and I was too afraid to get an NG tube and I wish I wasn’t because now I’m on an NG tube and wow, it’s way easier.

Food is foundational to social relations. It is part of how we label and categorize individuals, it can indicate group membership or culture, and it can demonstrate aspects of our personality; indeed, “what one eats, how one eats, when and with whom are guided by understandings of one’s identity within society” (Ochs et al., 1996, p. 8). Unsurprisingly, being restricted to exclusive enteral nutrition during school mealtimes is distressing for the participant and has implications for her sociability. Meals typically occur three times a day and consist of various foods that likely reveal something about you, as a person. For example, an individual’s food preferences may position them as health-minded or not, as an adventurous or picky eater, as a good cook (or from a home with a good cook), and so forth. It may also identify one’s cultural belonging or background, whether one wanted to disclose that information or not (e.g.,, children with packed lunches that signal particular geographic locations or cultural identifiers might hesitate to reveal food that is different to what their peers bring/eat). For those from specific cultural backgrounds, food can position them as other, thus contributing to or heightening preexisting feelings of difference and exclusion. The participant’s Modulen™ -based diet and the frequency in which she had to drink the formula identifies her as being different and would likely bring many questions from her peers both in class and during mealtimes. The social implications of her meals are simply too much; unlike the previous participant who continues to socialize despite his restrictions, this participant elects to leave school to avoid the issue altogether.

## Discussion

The participants articulated experiences of IBD that were complex, multi-faceted and that influenced several life domains. For some, the period leading up to diagnosis was replete with challenges that were unique to children and difficult to make sense of; for others, issues with sociability or medication side effects predominated. While our findings broadly align with those of existing research on children’s experiences of IBD, they also indicate some important nuances.

Kirk and Hinton (2019) point out that research exploring the diagnosis experience of children with chronic illness has been comparatively limited; indeed, the challenges associated with obtaining and making sense of a pediatric IBD diagnosis are underappreciated in the scholarly literature. This is unfortunate, as exploring our participants’ experiences of diagnosis has revealed important insights. Many participants described having a difficult time securing a diagnosis, which they mostly attributed to family physicians being unfamiliar with pediatric IBD symptoms. Participants often described a cycle of making trips to the hospital for testing and medical appointments only to either leave with their questions unanswered, or with a misdiagnosis. Of course, this experience is not particular to IBD; children with multiple sclerosis also describe the process of obtaining a diagnosis as being drawn out and confusing (Kirk & Hinton, 2019). We are unaware of any qualitative studies that explore the diagnostic journey, but our own analysis demonstrates that it is fraught with challenges unique to youths.

Having to undergo protracted periods of medical testing produced feelings of uncertainty, unknowing, frustration and difference; moreover, enduring this process was described as impelling maturation, or conversely, as denying participants a conventional childhood. Participants who lacked a formal diagnosis, but who required accommodations for their condition, were generally treated with suspicion; this was especially evident with unsympathetic school officials who resisted or outright denied them. While the work of Moody and colleagues’ (1999) demonstrates that teachers are often unknowledgeable, and therefore unsympathetic, about CD, our study demonstrates that this lack of sympathy extends even into the time period before diagnosis. As “the absence of a disease diagnosis can make an illness appear morally ambiguous” (Sharpe & Greco, 2019, p. 184), the phase prior to diagnosis was marked by concerns of morality. In these instances, the participant is forced to prove that their condition is “genuine” and that they merit receiving accommodations generally reserved for those who are “sick” (see Sharpe & Greco, 2019, p. 184). Thus, it is not surprising that some participants expressed a preference for being homeschooled before they were diagnosed. This finding sheds new light on school absences with respect to pediatric IBD. Other Canadian qualitative studies have reported that missing school is one of the most challenging and distressing aspects of pediatric IBD (Kluthe et al., 2018); however, our work complements these findings by pointing out that in the absence of a formal diagnosis (or when the social implications are perceived as being too great), school absences can be recognized as a welcomed relief.

Once diagnosed, our participants described several challenges related to understanding what it meant to have IBD. Other qualitative studies have demonstrated that children felt relieved upon diagnosis; however, this reaction differed based on how much they knew about the condition beforehand (Kluthe et al., 2018). Our own participants’ experiences demonstrate that feelings about diagnosis can perhaps be even more complex than has been acknowledged. Not only did they have to first understand what it meant to have IBD, but they further had to make sense of what it meant to be known as a child or adolescent with a disease, and what it meant for their identity and self-worth. Scholars have elsewhere noted that pediatric patient education typically does not attend to this important aspect of illness. In Morsa and colleagues’ (2018) qualitative study, participants were critical of the fact that patient education mostly centered around learning self-care skills for symptom management, which some felt simply reinforced the idea that they were “sick.” The participants further lamented that important existential questions (e.g., “Why me?“) were insufficiently addressed by parents or caregivers, which the youths saw as being necessary to address *prior* to learning self-care. Thus, it seems that there are two levels of diagnosis that require attention, both information on the disease itself and how to manage it, as well as how to make sense of the significance of disease, particularly as youths.

Children have described the side effects of their medication as being more grueling and more notable than the symptoms themselves (Nicholas et al., 2007); indeed, our participants likewise spoke at great lengths of the changes that their medications engendered. These included changes in body, behavior, motivations, goals, and attitudes, as well as unwelcomed sacrifices with respect to school, sports, goals/dreams, activities, and hobbies. As noted, one way that participants responded was to employ discursive strategies to make sense of these changes and to manage the implications they had for their identity. These strategies permitted the participants to save face and avoid being associated with the undesirable behaviors caused by their symptoms and treatment (e.g., overeating, gaining weight, not performing well in school, and no longer being athletic), thereby allowing them, in some cases, to bypass the need to renegotiate their identity, at least in the short-term. This is similar to the participants in Dibley’s et al., (2019) study where youths had to learn to differentiate between “being” and “having” a disease (e.g., *my disease* is disgusting versus *I* am disgusting) (Dibley et al., 2019).

ptIdentity changes related to ideas about the future were also salient. Participants described having to reluctantly put aside their dreams in favor of more attainable goals. Fourie and colleagues’ (2018) review noted that children and adolescents often feared their disease would prevent them reaching their full potential, as imagined by them before they were diagnosed. In contrast to some of the participants who viewed school absences as a welcomed relief, others regarded missed time from school with great anxiety, especially if that time away was perceived to imperil their scholastic performance, ability to graduate or to lead to fewer or poor career choices. The participants in Nicholas and colleagues’ (2007) study also described fearing that IBD would force them to choose a career that accommodates their condition, as opposed to something they enjoy.

Bodily changes produced by medication were clearly difficult to make sense of and carried implications for the participants’ gendered identity. Only one other study that we know of has addressed the fact that youths’ perceptions of their IBD-related bodily changes are shaped by gender. Nicholas and colleagues (2007) noted that girls were noticeably more concerned about issues related to weight gain, whereas boys tended to focus on growth delays and height. While the literature has often focused on bodily changes with respect to embarrassment and teasing (see Brydolf & Segesten, 1996), it is important to flesh out the gendered nature of this embarrassment.

In our third theme, we explored issues related to sociability, especially with respect to important aspects of childhood and adolescence—parties and meals with peers. In other studies, participants described feeling as though their condition forced them to miss out on important aspects of childhood or adolescence (see Nicholas et al., 2007). In some instances, our participants elected merely to avoid the activity altogether, such as when participants avoided sharing meals with friends or when they sought out homeschooling to make mealtimes easier. In this sense, our findings complement Sammut et al., (2015) study with older young adults who simply chose to avoid going out to bars or partake in other social activities to mitigate the chances of experiencing IBD-related issues while out. However, our study also demonstrates that some youths continue with socializing, though resign themselves to the fact that their sociability will be noticeably different from their friends, especially because they cannot drink.

This was raised by several of our older participants. Not drinking alcohol has implications, especially because alcohol consumption is not only built into our culture as playing a key role in social functions, but it is often also culturally normative for teenagers to drink (Bartram et al., 2016); accordingly, rejecting alcohol in some settings (even if for health reasons) can mean that the individual is rejecting both the drink and that which the drink symbolizes—social solidarity, relationship building, shared experiences, celebration, fun, and so forth (Bartram et al., 2016)

Meals are typically considered to be a social time, with commensality described as being a key human experience (Mäkelä, 2009). Other studies have tended to focus on the distress associated with not being able to eat as one wishes, or the materiality of eating; for example, some participants have said, “No one realizes how much they like food until they cannot eat” or “I miss getting full when I eat” (Nicholas et al., 2007). However, our study demonstrates that the social meaning attached to mealtimes is also of great importance. Participants relayed feelings of anxiety when not able to join in at mealtimes with their peers. Not being able to eat the same food as peers or family members can be a significantly challenging experience, even more so amongst individuals from cultural backgrounds where there are cultural meanings attached to food and food sharing. In cultures where food plays an important social role, the avoidance of particular foods or social events in which food sharing is prominent results in feelings of isolation, exclusion, and difference among members of these groups (Alexakis et al., 2015).

Based on our analysis, newly diagnosed Canadian pediatric patients might benefit from resources that address the following: (1) helping patients to understand why their disease was difficult to diagnose, including why it took so long; (2) information that helps patients make sense of the time period leading up to diagnosis, including why school officials, family, friends or peers were perhaps unsympathetic toward their condition or denied/resisted accommodations; (3) not only what IBD entails and how to manage it, but additional information to help them make sense of what it means to have a disease as a young person; (4) understanding that their identity and goals may change as their disease progresses, including help navigating the grief that may ensue; (5) that understandings of bodily changes produced by symptoms or medication are gendered, which will affect patients differently; (6) how to meaningfully navigate social situations (e.g., parties, mealtimes, school environments) where choosing to partake in a restricted manner or not at all can carry social ramifications for the child or adolescent; and (7) cultural nuances in understanding and adjusting to pediatric IBD—addressing the psychosocial implications that might result from changes in diet, food sharing practices and social gatherings (e.g., what it might mean from a cultural perspective to not eat traditional foods, and the implications this might have regarding feelings of belonging).

### Limitations and Future Directions

This study focused on further exploring the nuances of a relatively local group of Canadian children and adolescents’ experiences. While our in-depth approach was a strength of the study, the situatedness of the participants’ experiences must, of course, be acknowledged. Importantly, there is notable overlap in our participants’ experiences with those described in other studies, hinting at the prospect for transferability; however, it is likely that there are subtleties of the participants’ experiences that are unique to their context (e.g., culture, geographical location, available treatments, health care system, school district, education level, financial coverages, and current societal representations and stigma associated with illness). Accordingly, our recommendations may be more appropriate in some cases than in others.

For future research, it is important to note that parents and siblings may be able to offer additional insights that did not occur to the participants to divulge, though matter very much to researchers. This was evident in our interviews where parents contributed further detail that the participant either could not remember or struggled to articulate. Future studies might consider interviewing family members individually, not only as a way to triangulate youths’ experiences, but to further consider how having a child with this condition impacts the family dynamic and their life experiences. We did not formally recruit parents as research participants but benefited greatly from the contextual information shared to supplement their child’s response. We therefore recommend that future studies consider recruiting parents as participants to allow for their experiences to be shared in tandem with their child’s experience. However, interviewing children and their parents together (unless specifically requested by the child) can come with both costs and benefits. For example, some of our participants mentioned being hesitant about participating in interviews with their parents present due to the concern that their parents would only worry more about what was revealed or that they would overdramatize their experience. As such, future research exploring the experience of the family as a unit, might consider separate interviews to ensure that youth feel safe sharing information as joint interviews might impact the type of information shared, thus undermining the research participation experience and the quality of the interviews. On the other hand, some of our participants explicitly asked for their parents to attend and likely felt more at ease to participate with them present. In fact, in some cases, parents prompted longer responses from the child, as they were often younger and a bit shy. With parents present, we moved away from yes/no responses toward more engaging conversations regarding the participant’s experiences. Lastly, given that some of the side effects of IBD medications have different social and psychological implications for girls than for boys (e.g., weight gain), future studies should attend to the ways in which different aspects of one’s identity, or markers of social location (e.g., gender, race, and class) shape illness-related experiences and anxieties.

## Conclusion

The goals of this study were twofold: (1) to achieve a nuanced understanding of a regional cohort of Canadian children and adolescents’ experiences of IBD that takes into account the situated nature of their illness experience, so that we can (2) provide suggestions on the development of resources for children awaiting diagnosis or who are newly diagnosed to help navigate this daunting, confusing time. While there has been some work undertaken with respect to pediatric IBD experiences, it is important that we continue to undertake research on this topic so that we can more fully appreciate the challenges that children face in their unique contexts. Our participants’ experiences complement the work of other qualitative studies, as it is evident that youths’ experiences are characterized by physical, emotional, and social challenges that are unique to their developmental stage. Our themes of *challenges related to diagnosis, managing identity and making sense of change,* and *navigating sociability* demonstrate that newly diagnosed pediatric patients require a holistic approach to treatment that attends not only to their physical well-being, but also their emotional and social needs.
